# *Loxosceles gaucho* Spider Venom: An Untapped Source of Antimicrobial Agents

**DOI:** 10.3390/toxins10120522

**Published:** 2018-12-06

**Authors:** Paula J Segura-Ramírez, Pedro I Silva Júnior

**Affiliations:** 1 Special Laboratory for Applied Toxinology (LETA), Butantan Institute, São Paulo CEP 05503-900, SP, Brazil; paula.segura@icb.usp.br; 2 Institute of Biomedical Sciences, University of São Paulo, São Paulo CEP 05508-900, SP, Brazil

**Keywords:** *Loxosceles*, venom, anionic antimicrobial peptides, antimicrobial resistance

## Abstract

The remarkable ability of microorganisms to develop resistance to conventional antibiotics is one of the biggest challenges that the pharmaceutical industry currently faces. Recent studies suggest that antimicrobial peptides discovered in spider venoms may be useful resources for the design of structurally new anti-infective agents effective against drug-resistant microorganisms. In this work, we found an anionic antibacterial peptide named U_1_-SCRTX-Lg1a in the venom of the spider *Loxosceles gaucho*. The peptide was purified using high-performance liquid chromatography (HPLC), its antimicrobial activity was tested through liquid growth inhibition assays, and its chemical properties were characterized using mass spectrometry. U_1_-SCRTX-Lg1a was found to show a monoisotopic mass of 1695.75 Da, activity against Gram-negative bacteria, a lack of hemolytic effects against human red blood cells, and a lack of cytotoxicity against human cervical carcinoma cells (HeLa). Besides this, the sequence of the peptide exhibited great similarity to specific regions of phospholipases D from different species of *Loxosceles* spiders, leading to the hypothesis that U_1_-SCRTX-Lg1a may have originated from a limited proteolytic cleavage. Our data suggest that U_1_-SCRTX-Lg1a is a promising candidate for the development of new antibiotics that could help fight bacterial infections and represents an exciting discovery for *Loxosceles* spiders.

## 1. Introduction

Despite the great advances made on the field of antibiotic therapy since the early 20th century, infectious diseases remain major causes of death in human population due to the great ability of microorganisms to develop resistance to conventional antibiotics, compromising their effectiveness. Because of this, it has become urgent to find novel sources of non-traditional antibiotics in order to develop new drugs effective against pathogenic microorganisms [1].

In this regard, antimicrobial peptides (AMPs) emerge as promising candidates for the control of infectious diseases due to their low resistance rates, potent activity, and unique mechanism of action [2,3]. These endogenous molecules, which are mostly genetically encoded, constitute a primitive immune defense mechanism present in the vast majority of living organisms: viruses, bacteria, plants, insects, fishes, amphibians, and mammals [4]. This group of peptides exhibits a broad range of biological properties, from the direct neutralization of cancer cells and invading pathogens such as bacteria, fungi, viruses, and protozoan parasites, to the modulation of the immune response of the host [5,6,7].

The venom of spiders contains pore-forming peptides whose main purpose is to depolarize cell membranes and damage the tissues of their prey [1]. However, some remarkable examples have shown that these peptides also may play a role as genuine antimicrobials by protecting the spider against the potential invasion of infectious organisms arising from the injection of venom into their prey [8,9,10]. Consequently, in recent years, the idea of using AMPs discovered in spider venoms as tools for the design of structurally novel drugs has received much attention from experts in the field of biotechnology and the pharmaceutical industry [11], leading to the identification and study of several of these molecules [12,13,14,15,16,17,18]. Among these peptides, Juruin shows a lack of hemolytic activity against human blood cells [13], while LyeTx I and Lycosin-II display a weak hemolytic activity in high concentrations [16,18]. In the case of CIT 1a, a negligible effect on cell viability was observed [14].

Brown spiders (the *Loxosceles* genus) are members of the select group of the world’s most venomous spiders. Their bite causes a condition called loxoscelism and has two clinical manifestations: cutaneous and systemic, which take place approximately in 83.3 and 16.7% of cases, respectively [19]. The first and most frequent manifestation is associated with necrotic skin lesions, which advance gradually from the bite. On the other hand, at the systemic level, it is common to observe weakness, fever, nausea and vomiting, hematuria, pruritic reactions, renal failure, jaundice hemoglobinuria, and disseminated intravascular coagulation [20,21]. Due to the prevalence of loxoscelism as a public health problem in several South American countries such as Argentina, Peru, Chile, and Brazil, brown spider venoms have been studied in order to increase the knowledge about the pathophysiology of loxoscelism [22]. Although the complete content of the venom is not yet fully understood, many investigations have shown that it consists of a complex mixture of proteins and peptides with a molecular mass profile in the range of 2 to 40 kDa. These components have toxic and/or enzymatic activities and act synergistically [20,21,22,23,24]. To date, many brown spider toxins have been described and their corresponding biochemical properties have been characterized, providing key information about their great potential for biotechnological purposes such as the design of pharmacological tools, diagnostic and immunotherapeutic reagents, cytotoxicity inducers, and biopesticides [20,21,25]. Included among these molecules are phospholipases D [26,27,28,29,30,31,32,33,34,35], astacins (metalloproteases) [36,37,38,39,40,41], hyaluronidases [42,43,44,45,46], serine proteases [44,47,48,49,50], translationally controlled tumor protein (TCTP) [47,48,51], and inhibitor cystine knot (ICK) peptides [24,52,53].

Being aware of the antibiotic resistance problem, through the present work we aim to generate data that may lead to the creation of new potential drugs effective against pathogenic microorganisms. Furthermore, this work has the additional objectives of increasing the literature about AMPs from *Loxosceles* spiders and setting up a basis for future studies directed to elucidate new modes of action of these molecules. Herein, we present the characterization of a novel AMP isolated from *Loxosceles gaucho* venom.

## 2. Results and Discussion

### 2.1. Purification of U_1_-SCRTX-Lg1a from the Venom of L. gaucho

**Figure 1:**
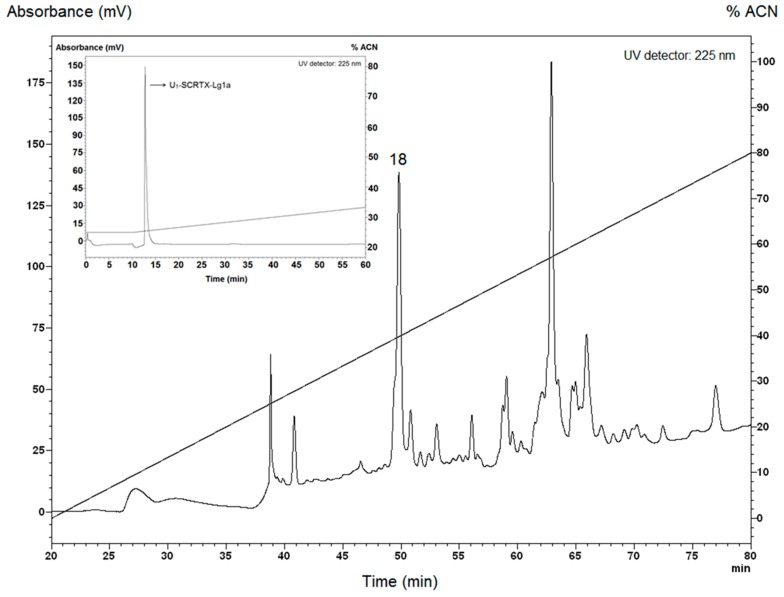
Purification of U_1_-SCRTX-Lg1a from the crude venom of *L. gaucho* by RP-HPLC. The acidified venom sample was analyzed on a semi-preparative column Jupiter C18 with a linear gradient from 0% to 80% ACN in acidified water at a flow rate of 1.5 mL/min over 60 min. The numbered peak (18) corresponds to the fraction that showed antimicrobial activity and was eluted at 49.1–50.6 min. Fraction 18 was re-chromatographed on the same system and run from 26% to 46% ACN in acidified water over 60 min. The peak indicated with an arrow corresponds to the U_1_-SCRTX-Lg1a fraction.

The crude venom of *L. gaucho* was collected by electrostimulation. The first stage of venom separation was performed by reversed-phase high-performance liquid chromatography (RP-HPLC), which resulted in isolation of at least 32 different fractions that were analyzed in liquid growth inhibitory assays using *Escherichia coli* SBS363, *Micrococcus luteus* A270, *Aspergillus niger*, and *Candida albicans* MDM8. The antimicrobial activity occurred in the fraction 18 eluted with retention times of 49.1–50.6 min (Figure 1), which was effective only against *E. coli* SBS363, a Gram-negative bacterium. This fraction was further applied to the same C18 RP-HPLC column for the purification of individual compounds. Among these, only one had a pronounced antibacterial activity: U_1_-SCRTX-Lg1a (Figure 1). This name was established according to the nomenclature indicated by King et al. [54]. The fraction was quantified based on absorbance at 205 nm and its final concentration in 500 µL of ultrapure water was 23 µM (38 µg/mL).

This finding represents an exciting new source of information for the production of antimicrobial drugs and contributes to the limited existing literature on antimicrobial molecules from the venom of *Loxosceles* spiders. This fact is quite surprising because the venom of these organisms has been well-studied due to its medical importance and several of the toxins that compose it have been characterized, providing new information about the pathophysiology of envenomation and the biotechnological potential of these molecules [20,21]. However, so far there is only one report that indicates that *L. gaucho* venom contains low-molecular-mass molecules with antimicrobial activity against *Pseudomonas aeruginosa*. The study also mentions that the whole venom of this spider does not influence the proliferation of *P. aeruginosa*, but increases its biofilm formation, as well as the production of gelatinase and caseinase [55].

### 2.2. Antimicrobial Activity and Minimum Inhibitory Concentrations (MICs)

U_1_-SCRTX-Lg1a was assessed for antimicrobial activity against five species of Gram-negative bacteria, three species of Gram-positive bacteria, one species of fungus, and two species of yeast (Table 1). All Gram-negative bacteria species tested showed sensitivity to U_1_-SCRTX-Lg1a, which was active at a concentration range between 1.15 μM (1.9 μg/mL) and 4.6 μM (7.6 μg/mL). *P. aeruginosa* ATCC 27853 was the most sensitive to the fraction, with an MIC of 1.15 μM (1.9 μg/mL). U_1_-SCRTX-Lg1a was not effective against Gram-positive bacteria, fungus, or yeast at the concentrations investigated (Table 1).

Interestingly, U_1_-SCRTX-Lg1a has lower MICs against the Gram-negative bacterial strains tested than those of Lacrain, an AMP found in the body extract of centipede *Scolopendra viridicornis* previously identified by our group [56]. On the other hand, compared to Gomesin, a potent host defense peptide isolated from the hemocytes of the spider *Acanthoscurria gomesiana* [57], the U_1_-SCRTX-Lg1a MICs against *E. coli* SBS363 and *E. coli* D31 are higher; however, it has a slightly more pronounced antimicrobial effect against *P. aeruginosa* ATCC 27853 and *Enterobacter cloacae* β-12. It is worth noting that *P. aeruginosa* PA14, a hyper-virulent burn wound isolated strain, was also sensitive to U1-SCRTX-Lg1a.

Because of the antibacterial activity described, we believe that U_1_-SCRTX-Lg1a could have Gram-negative specificity. Under this assumption, it can be said that this fraction has a great therapeutic potential, considering that recently identified AMPs that were introduced into clinical practice mainly display activity against Gram-positive bacteria while being ineffective against Gram-negative organisms due to their use of multiple mechanisms that work synergistically to resist AMPs [58].

Nonetheless, it would be impelling to investigate other biological activities of this fraction in order to have a clearer notion of all its biotechnological potential, since other molecules with several properties are constantly reported in the literature. For example, spinigerin, a 25-amino acid peptide obtained from the fungus-growing termite *Pseudacanthotermes spiniger*, exhibits antibacterial, antifungal, and antiviral properties, and recently it was also found that spinigerin induces apoptosis-like cell death in *Leishmania donovani* [59].

### 2.3. Hemolytic Activity

**Figure 2:**
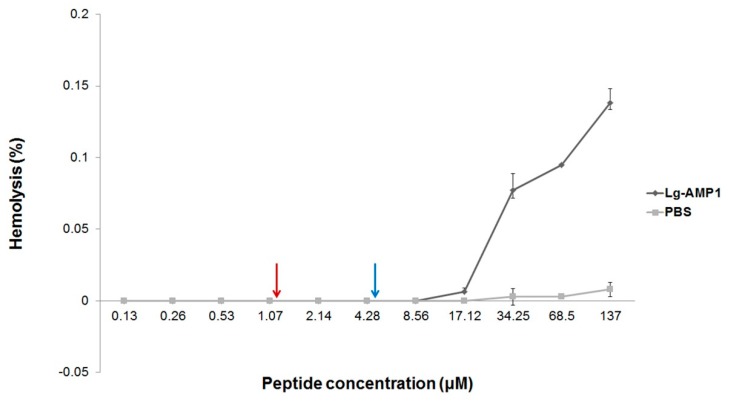
Hemolytic effects of U1-SCRTX-Lg1a on human erythrocytes. The concentration-response curve of hemolytic activity of the peptide shows its extremely low toxicity even at the maximum concentration tested (137 µM), which is very similar to the negative control (phosphate-buffered saline (PBS)). The two arrows (red and blue) indicate the concentration range in which the fraction showed antimicrobial activity. The results represent the mean ± standard deviation of three independently developed experiments.

To determine the effect of U_1_-SCRTX-Lg1a on human erythrocytes at the antimicrobial concentrations, its hemolytic activity was tested. After incubating red blood cells from a healthy donor with the fraction up to a concentration of 137 μM for 3 h at 37 °C, no hemoglobin release was observed, indicating that U_1_-SCRTX-Lg1a does not cause lysis of human erythrocytes within these concentrations (Figure 2). These data suggest that the mechanism of action of U_1_-SCRTX-Lg1a does not involve the disruption of cell membranes and that this fraction is not part of the group of toxins responsible for the hemolytic properties ascribed to the whole venom of *Loxosceles* spiders [21].

For antimicrobial molecules to be interesting for systemic applications, they must show low toxicity against erythrocytes [60]. Taking this into account, U_1_-SCRTX-Lg1a emerges as a promising template for the development of novel antibiotics.

### 2.4. Cytotoxicity of U_1_-SCRTX-Lg1a

**Figure 3:**
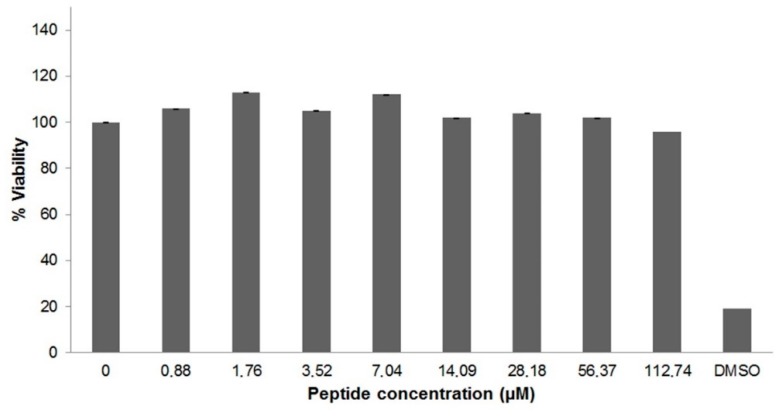
Cytotoxicity of U1-SCRTX-Lg1a. HeLa cells were incubated with various concentrations (0.88 μM to 112.74 μM) of the fraction for 48 h at 37 °C. Effects on cell viability were determined by performing an MTT assay. Untreated HeLa cells were used as a negative control and HeLa cells treated with DMSO served as a positive control. The results correspond to the mean ± standard deviation of three experiments carried out independently.

The cytotoxicity of U_1_-SCRTX-Lg1a against human cervical carcinoma cells (HeLa) was assessed using the 3-(4,5-dimethylthiazol-2-yl)-2,5-diphenyltetrazolium bromide (MTT) assay. After incubating the cells with the fraction at various concentrations (0.88 μM to 112.74 μM), it was observed that the amount of formazan produced by the mitochondria of living cells did not vary considerably, which indicates that U1-SCRTX-Lg1a did not affect HeLa cell viability even at a high concentration of 112.74 μM (Figure 3). This lack of cytotoxicity presented by the fraction against HeLa cells at the antimicrobial concentration range suggests a possible specificity of U1-SCRTX-Lg1a against bacteria, leading us to consider this as an indicator of its safety for the development of antibiotics for mammalian organisms.

### 2.5. U_1_-SCRTX-Lg1a Identification

**Figure 4:**
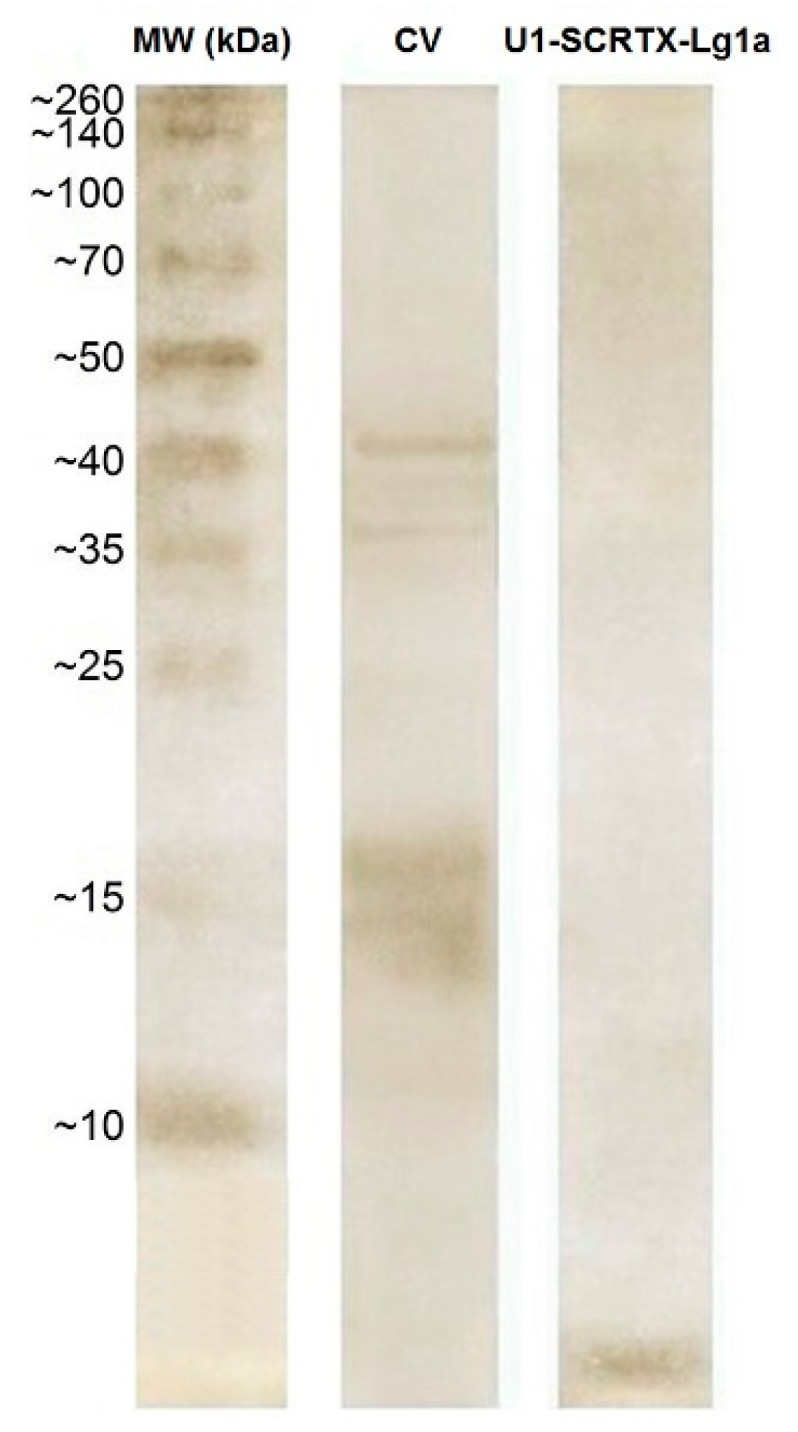
Electrophoretic analysis of U_1_-SCRTX-Lg1a. Silver-stained 12% SDS-PAGE gel of the crude venom of *L. gaucho* (CV) (5 µg) and purified antimicrobial U_1_-SCRTX-Lg1a (2.5 µg) under non-reducing conditions. On the left are numbers that correspond to the positions of molecular weight markers (MW) expressed in kDa.

The silver-stained 12% SDS-polyacrylamide gel electrophoresis (SDS-PAGE) analysis did not show bands corresponding to the purified antimicrobial U_1_-SCRTX-Lg1a within the molecular weight range established by the marker (MW); instead, it was observed that the fraction accumulated near the bottom of the gel, while other protein components of *L. gaucho* crude venom (CV) whose molecular weights are higher than 10 kDa did appear on the gel and were clearly separated (Figure 4). Through this, we inferred that U_1_-SCRTX-Lg1a has a molecular weight lower than 10 kDa.

**Figure 5:**
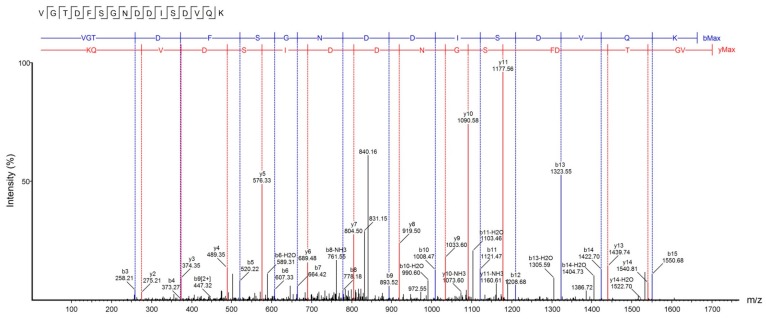
Collision-induced dissociation (CID) spectrum of the de novo sequenced antimicrobial peptide, U1-SCRTX-Lg1a. The ions belonging to -y (red) and -b (blue) series indicated in the spectrum correspond to the amino acid sequence of the peptide: VGTDFSGNDDISDVQK. The fragments of the sequenced peptide are represented by standard amino acid code letters.

**Figure 6:**
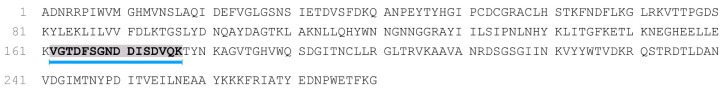
Peptide spectrum match indicated by a blue line below the sequence of the phospholipase D LgRec1. U_1_-SCRTX-Lg1a covered 6% of the whole protein sequence.

Accurate molecular mass and sequence of U_1_-SCRTX-Lg1a were established by tandem mass spectrometry (MS/MS) data interpretation using PEAKS Studio software (v8). The fragmentation pattern revealed a peptide sequence of 16 amino acids (VGTDFSGNDDISDVQK) with a monoisotopic mass of 1695.75 Da and an average local confidence (ALC) of 88% (Figure 5). The U_1_-SCRTX-Lg1a database searches performed through the PEAKS DB tool revealed that this native peptide fraction of *L. gaucho* venom may be derived from the phospholipase D LgRec1 [33], covering 6% of the whole protein sequence (Figure 6).

To our knowledge, U_1_-SCRTX-Lg1a is the first antimicrobial peptide isolated from the venom of *L. gaucho*, an araneomorph spider that belongs to the Sicariidae family. Before this study, the only peptides reported for *Loxosceles* spiders were ICKs [24,52,53], a family of structural peptides with several cysteine residues that form disulfide bonds that result in a knot, which have generated great interest due to their ability to specifically bind to insect ion channels, conferring them great potential for the development of efficient bioinsecticides for the control of pests that can affect the agricultural sector or insects vectors of infectious diseases [20,21]. Considering this, it is safe to say that U_1_-SCRTX-Lg1a is the newest member of the group of bioactive peptides isolated from the venom of *Loxosceles* spiders. As such this complex protein mixture invites a new biotechnological approach in view that it may represent a valuable alternative to standard antimicrobial therapies as a new and non-conventional anti-infective agent. 

### 2.6. Structure and Physicochemical Characteristics of U_1_-SCRTX-Lg1a

**Figure 7:**
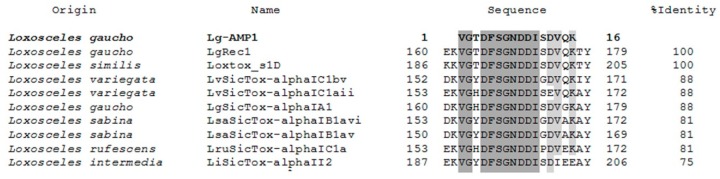
Multiple alignment analysis of the deduced amino acid sequence of U_1_-SCRTX-Lg1a from *L. gaucho* with specific fragments of phospholipases D previously reported from different species of *Loxosceles* spiders. Regions that show identical amino acids among all species are shaded in dark gray and those strongly conserved are shaded in light gray. Sequences alignment was performed with Clustal Omega (http://www.ebi.ac.uk/Tools/msa/clustalo/; accessed on 25 September 2018) and modified manually.

Sequence similarity searches with the Basic Local Alignment Search Tool (BLAST) allowed us to perform a multiple alignment analysis of the amino acid sequence of U_1_-SCRTX-Lg1a using the Clustal Omega program. BLAST searches and sequence alignment exhibited the peptide homology to specific regions of nine phospholipases D found in the venom of *Loxosceles* spiders (Figure 7). The amino acid sequences of phospholipases D LgRec1 (fragments 162–177) and Loxtox_s1D (fragments 188–203) had the highest homology to U_1_-SCRTX-Lg1a, showing a 100% identity match with the peptide. On the other hand, LvSicTox-alphaIC1bv (fragments 154–169), LvSicTox-alphaIC1aii (fragments 155–170), LgSicTox-alphaIA1 (fragments 162–177), LsaSicTox-alphaIB1avi (fragments 155–170), LsaSicTox-alphaIB1av (fragments 152–167), and LruSicTox-alphaIC1a (fragment 155–170) had a lower but still significant homology to the peptide with an identity match range between 88–81%. Finally, LiSicTox-alphaII2 had the lowest homology to U_1_-SCRTX-Lg1a, exhibiting a 75% identity match. The alignment also showed that all analyzed sequences contain two regions with identical amino acids: one of two residues (VG) and another one of eight residues (DFSGNDDI). Furthermore, three positions are strongly conserved and two are occupied by residues of functional similarity (D and E, both with negatively charged R groups; V and I, both with nonpolar R groups), indicating that these positions represent conservative amino acid exchanges [61].

The venoms of several species are rich sources of antimicrobial molecules, and it has been suggested that the presence of these molecules is useful for organisms like spiders and scorpions to clean the biological conducts that transport venom from the gland where it is produced to the tip of the venom injector, protecting them from potential infections [1]. Based on this, the PEAKS DB searches and resulting multiple sequence alignment led us to hypothesize that, as has been previously reported in studies on AMPs derived from large proteins found in the venom of spiders and snakes [12,62], U_1_-SCRTX-Lg1a may have originated from a limited proteolytic cleavage after K residues suffered from the phospholipase D LgRec1 previously isolated from the venom of *L. gaucho* [33], suggesting that these dermonecrotic toxins may be important factors in the immune response of *Loxosceles* spiders by functioning as a substrate for the generation of AMPs.

Some physical and chemical characteristics of U_1_-SCRTX-Lg1a were predicted by performing a sequence analysis using the ProtParam tool available on the bioinformatics resource portal ExPASy. The net charge, grand average of hydropathicity (GRAVY), and theoretical isoelectric point (pI), among other properties, were calculated (Table 2). U_1_-SCRTX-Lg1a is an anionic molecule (net charge of −3) due to the presence in its structure of four aspartic acid residues (D), a negatively charged amino acid, and just one lysine residue (K), which is a positively charged amino acid. Furthermore, the peptide has a pI of 3.77, indicating that this is the pH value at which its net charge is equal to 0. Additionally, the GRAVY score, a metric of the overall hydrophobicity/hydrophilicity of polypeptides, was in the negative range for U_1_-SCRTX-Lg1a (−0.769), showing that this AMP could be hydrophilic in nature.

Most AMPs are cationic, which facilitates their interaction with anionic microbial membranes, leading to a variety of effects that includes membrane permeabilization, depolarization, leakage, and lysis, which ends in cell death. However, many evidences indicate that some cationic AMPs (CAMPs) can interact with intracellular anionic targets such as DNA, RNA, or cell wall components whilst others appear to have immunomodulatory properties [63]. Generally, CAMPs also have an isoelectric point close to 10, which is very similar to detergents and is consistent with the membrane-disruptive mechanisms of action proposed for many of these peptides [64]. Taking this into account, the net negative charge of U_1_-SCRTX-Lg1a and its low pI indicate that this peptide is part of the interesting group of anionic antimicrobial peptides (AAMPs), which have been considered as an essential part of the innate immunity of vertebrates, invertebrates, and plants [63].

Because some microbial strains either possess inherent resistance to CAMPs or can develop resistance to these molecules, a rapidly growing number of AAMPs have been identified in recent years, which appear to have evolved to counter microbes with resistance to CAMPs and would thus seem well-suited for the development of novel antimicrobial agents [65]. The interaction with the membrane appears to be crucial for the antimicrobial mechanisms of AAMPs, which include toroidal pore formation and the Shai–Huang–Matsazuki model of membrane interaction along with membranolysis via tilted peptide formation and pH-dependent amyloidogenesis [66]. However, it has also been reported that some of these AAMPs use non-membranolytic mechanisms of action that and involve the binding of metal ions through the residues of aspartic acid and glutamic acid to form cationic salt bridges with negatively charged components of microbial membranes. This action would allow the translocation of these peptides across the cell membrane into the cytoplasm, where they could act on internal cellular targets [63]. Taking into consideration that the results of the hemolytic assay suggest a non-membrane disruptive mechanism of action for U_1_-SCRTX-Lg1a, it would be interesting to elucidate the way in which this anionic peptide interacts with its target microorganisms in future studies.

**Figure 8:**
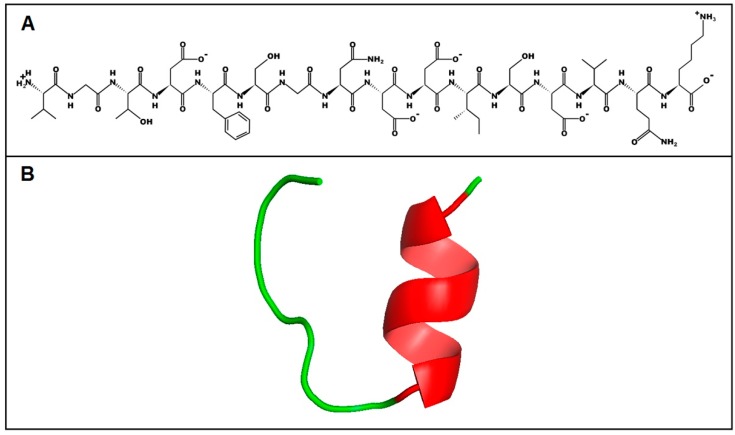
Structure prediction of U_1_-SCRTX-Lg1a. (**A**) Primary structure of U_1_-SCRTX-Lg1a generated by the PepDraw tool. (**B**) Secondary structure of U_1_-SCRTX-Lg1a as predicted by the online I-TASSER server.

To predict the primary and secondary structure of the peptide from its amino acid sequence, the PepDraw tool and I-TASSER server were used, respectively (Figure 8). The predicted model by I-TASSER suggests that U_1_-SCRTX-Lg1a tends to form an α-helix between the ISDV residues and that the rest of the structure is a coil, which implies an absence of regular secondary structure in the largest portion of the molecule. The C-score, a measure of confidence that allows an estimation of the quality of the predicted structure, was −0.77 for this peptide, indicating a correct global topology for the model.

## 3. Conclusions

In summary, we isolated, purified, and characterized a native AAMP from the venom of the spider *L. gaucho*, named U_1_-SCRTX-Lg1a. The 1695.75 Da peptide shows a potent and rapid antibacterial effect on different strains of Gram-negative bacteria, a lack of hemolytic activity against human erythrocytes, a lack of cytotoxicity against HeLa cells, and a remarkable similarity to specific regions of phospholipases D from different species of *Loxosceles* spiders. These data suggest that U_1_-SCRTX-Lg1a have potential for the design of novel and non-conventional therapeutic agents effective against infectious diseases caused by bacteria. Thus, the mechanism of action of U_1_-SCRTX-Lg1a should be determined in future studies. Finally, it is worth highlighting that this is the first peptide with antimicrobial properties described for the *Loxosceles* genus, whose venom has been extensively studied, indicating that this complex mixture of proteins and peptides could be a relatively untapped source of antimicrobial molecules with novel mechanisms of action.

## 4. Materials and Methods

### 4.1. Microbial Strains

Bacterial and fungal strains were obtained from the collection of microorganisms of the Special Laboratory for Applied Toxinology (LETA) of the Butantan Institute (São Paulo, Brazil). Among these strains were: *E. coli* SBS363, *E. coli* D31, *P. aeruginosa* ATCC 27853, *P. aeruginosa* PA14, *E. cloacae* β-12, *M. luteus* A270, *Staphylococcus aureus* ATCC 29213, *Bacillus subtilis* ATCC 6633, *C. albicans* MDM8, *C. krusei* IOC 4559, and the filamentous fungus *A. niger* isolated from bread.

### 4.2. Animals

**Figure 9:**
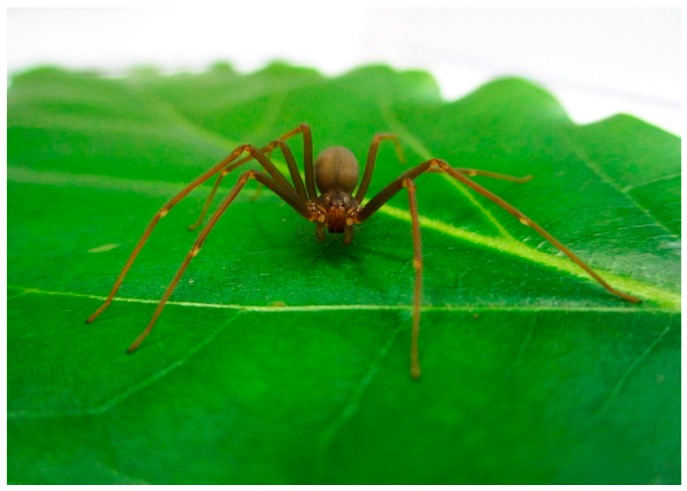
*L. gaucho* adult female (Araneae, Sicariidae).

Adult specimens of *L. gaucho* were collected and kept alive in the bioterium of the LETA (Figure 9). The animals were collected under the Permanent License for the Collection of Zoological Material no. 11024-3 provided by the Brazilian Institute of Environment and Renewable Natural Resources (IBAMA) and Special Authorization for Access to Genetic Patrimony no. 001/2008.

### 4.3. Venom Fractionation and U_1_-SCRTX-Lg1a Purification

**Figure 10:**
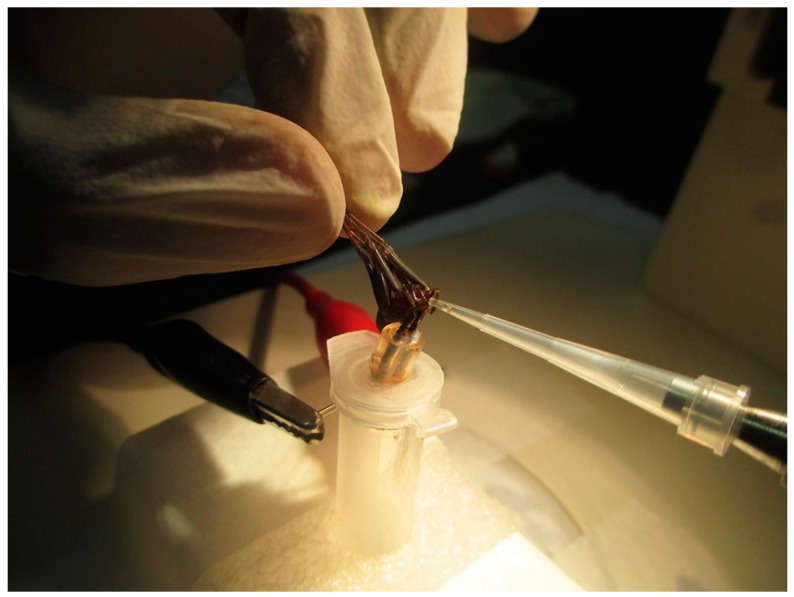
Venom collection of *L. gaucho* by electrostimulation. The spider was immobilized holding its legs back and released the venom into a 10-µL pipette tip. The venom was later pooled in a 0.5-mL Eppendorf tube.

After capture, the arachnids were kept in quarantine for seven days without food as a preparation for the extraction of venom. For this procedure, the technique performed was electrostimulation using an AVS-100 electric shock generator (AVS Projetos Especiais, São Paulo, Brazil), which consisted of applying brief 15 V electric shocks repeatedly to the chelicerae of the spiders until they released the venom (Figure 10). *L. gaucho* crude venom was collected with a micropipette, pooled in a 0.5-mL Eppendorf tube that remained on ice throughout the extraction period, and then immediately freeze-dried and stored at −80 °C before being resuspended in acidified water (trifluoroacetic acid (TFA) 0.05%) for the RP-HPLC fractionation, which was carried out 18 hours later. The volume of crude venom collected was approximately 15 µL. The insoluble material was eliminated by centrifugation at 14,000× *g* for 2 min and the supernatant was directly subjected to RP-HPLC on a semi-preparative column Jupiter C18 (Phenomenex International, Torrance, CA, USA) equilibrated at room temperature with 0.05% TFA in ultrapure water. The purification of the sample was carried out using acetonitrile (ACN)/water/0.05% TFA gradients of 0% to 80% ACN at a flow rate of 1.5 mL/min over 60 min. Ultraviolet (UV) absorbance of the effluent was monitored at 225 nm. The eluted peak fractions were manually collected and were vacuum-dried before being used in antimicrobial activity assays. The fraction with antimicrobial activity (peak 18) was further purified using a linear gradient from 26% to 46% ACN at a flow rate of 1 mL/min for 60 min on the same column and it was quantified based on absorbance at 205 nm using a NanoDrop 2000 spectrophotometer (Thermo Fisher Scientific, Waltham, MA, USA). Peptide purity of the obtained individual component with antimicrobial activity (U_1_-SCRTX-Lg1a) was confirmed by mass spectrometry and amino acid sequencing.

### 4.4. Antimicrobial Assays

During the purification process, the antimicrobial effects of the fractions were evaluated by liquid growth inhibition assays against *E. coli* SBS363, *M. luteus* A270, *A. niger*, and *C. albicans* MDM8. Bacteria were cultured in poor nutrient broth (PB) (1.0 g peptone in 100 mL of water containing 86 mM NaCl at pH 7.4; 217 mOsm), and the fungus and yeasts were cultured in poor potato dextrose broth (1/2-strength PDB) (1.2 g potato dextrose in 100 mL of water at pH 5.0; 79 mOsm). Determination of antimicrobial activity was executed using a five-fold microtiter broth dilution assay in 96-well sterile plates (Shanghai Beiyi Bioequip Information Co., Ltd., Shanghai, China) at a final volume of 100 μL. Mid-log phase cultures were diluted to a final concentration of 5 × 10^4^ CFU/mL for bacteria and 5 × 10^5^ CFU/mL for the fungus and yeasts [67,68]. Dried fractions were dissolved in 200 μL of ultrapure water and then 20 μL were aliquoted into each well with 80 μL of microbial dilution. The assays were carried out in duplicate. Sterile water and PB or PDB were used as controls. Tetracycline was also used as a growth inhibition control. The microtiter plates were incubated for 18 h at 30 °C. Growth inhibition was determined by measuring absorbance at 595 nm.

### 4.5. Concentration of U_1_-SCRTX-Lg1a

The concentration of U1-SCRTX-Lg1a was determined through the Lambert-Beer law using the molar extinction coefficient at 205 nm absorption [69], obtained using the Protein Parameter Calculator tool available at the Nick Anthis website (http://nickanthis.com/tools/a205.html; accessed on 25 September 2018).

### 4.6. MICs of U_1_-SCRTX-Lg1a

The MICs were established using the purified fraction U_1_-SCRTX-Lg1a against Gram-negative bacterial strains, Gram-positive bacterial strains, a fungal strain, and a yeast strain, as described above (Section 4.1). The fraction dissolved in ultrapure water at a final concentration of 23 μM was used to perform serial dilutions in 96-well sterile plates at a final volume of 100 μL. For that, 20 μL of the fraction was applied to each well at a serial dilution of two-fold microtiter broth dilution and added to 80 μL of the microbial dilution (modified from Ehret-Sabatier et al. [70]). Microbial growth was measured by monitoring the increase in optical density at 595 nm using a Victor 3TM 1420 multilabel counter (PerkinElmer, Waltham, MA, USA). The MIC was defined as the lowest concentration without visible growth after incubation at 30 °C for 18 h. Assays were performed in duplicate.

### 4.7. Determination of Hemolytic Activity

The hemolytic activity of the U_1_-SCRTX-Lg1a fraction was assessed against human erythrocytes from a healthy adult donor. A 3% (*v*/*v*) suspension of washed erythrocytes in 0.15 M phosphate-buffered saline (PBS) was incubated with U_1_-SCRTX-Lg1a at concentrations ranging from 0.13 μM to 137 μM in a U-bottom 96-well plate for 3 h at 37 °C with intermittent shaking. The supernatants were first collected and transferred to a flat-bottom 96-well plate, and then hemolysis was determined by measuring the absorbance of each well at 414 nm in a FlexStation 3 multi-mode microplate reader (Molecular Devices, San Jose, CA, USA). Assays were conducted in triplicate. The hemolysis percentage was expressed in relation to a 100% lysis control (erythrocytes incubated with 0.1% Triton X-100), PBS was used as a negative control, and the calculation was made according to the following equation: % hemolysis = (A_sample_ − A_negative_)/(A_positive_ − A_negative_).

### 4.8. Cytotoxicity Assay against HeLa Cells

The toxicity of the fraction U1-SCRTX-Lg1a against HeLa cells was evaluated using the MTT colorimetric assay to measure cell viability. First, the cells were cultivated and maintained in DMEM culture medium, supplemented with 10% heat-inactivated calf serum. After that, the HeLa cells were seeded in 96-well sterile plates (2 × 10^5^ cells/well) and cultured for 24 h at 37 °C in a humidified atmosphere containing 5% CO_2_. Serial dilutions of the fraction were carried out using DMEM to obtain final concentrations ranging from 0.88 μM to 112.74 μM, which were added and allowed to react with the cells for 48 h, followed by the addition of 20 μL MTT (5 mg/mL in PBS) for another 4 h at 37 °C. Finally, 150 μL of isopropanol were added to dissolve the formazan crystals. Absorbance at 550 nm was measured and assays were conducted in triplicate. Cell survival was calculated according to the following equation: % survival = (A_treated cells_/A_untreated cells_) × 100.

### 4.9. SDS-PAGE Analysis

Samples of *L. gaucho* crude venom (5 µg) and U_1_-SCRTX-Lg1a (2.5 µg) were analyzed by 12% tris-glycine SDS-PAGE under non-reducing conditions [71], using a Spectra Multicolor Broad Range Protein Ladder (Thermo Fisher Scientific, Waltham, MA, USA) to estimate the molecular mass. Before the electrophoresis, the samples were solubilized in sample buffer. The total running time was 3 h at 120 V. After electrophoresis, the gel was stained with silver nitrate [72].

### 4.10. Mass Spectrometry Analysis and U_1_-SCRTX-Lg1a Identification

The fraction U_1_-SCRTX-Lg1a was analyzed in positive ion mode on an LTQ Orbitrap Velos mass spectrometer (Thermo Fisher Scientific, Bremen, Germany) coupled to an Easy-nLC II (Thermo Fisher Scientific, Bremen, Germany), according to Abreu et al. [12] with some modifications. The mass spectrometer was programmed for a full scan, recorded between *m*/*z* 300 and 2000 with a resolution of 60,000 (at *m*/*z* 400). The 10 most abundant peaks were fragmented using collision-induced dissociation (CID) and analyzed in an ion trap. The isolation window for precursor ions was set to 2 *m*/*z*, the minimum count of ions to trigger events (MS^2^) was 10,000, and the dynamic exclusion time was set to 90 s. Normalized collision energy was set to 35%. In order to identify the U_1_-SCRTX-Lg1a fraction, mass spectrometry (MS) raw data were processed and searched in the PEAKS Studio software (v8; Bioinformatics Solutions, Waterloo, ON, Canada) [73]. To determine its amino acid sequence, we performed de novo sequencing from MS/MS data with the following parameters: a precursor mass tolerance of 10 ppm and a fragment ion mass tolerance of 0.5. De novo peptides, whose ALC scores ≥80% were searched against the UniProt-SwissProt database using the PEAKS DB tool. The peptide false discovery rate (FDR) was predictable by the decoy fusion method and was selected at a maximum of 1%.

### 4.11. U_1_-SCRTX-Lg1a Analysis with Bioinformatics Tools

The resulting amino acid sequence of U_1_-SCRTX-Lg1a was submitted to searches for regions of local similarity against proteins from arthropods registered on the public database provided at the National Center for Biotechnology Information (NCBI) website using BLAST (https://blast.ncbi.nlm.nih.gov/Blast.cgi; accessed on 25 September 2018). The physicochemical parameters of the sequence were calculated using the ProtParam tool available on the bioinformatics resource portal ExPASy of the Swiss Institute of Bioinformatics (SIB) website (http://web.expasy.org/protparam/; accessed on 25 September 2018). Finally, the potential primary structure of U_1_-SCRTX-Lg1a was generated using the PepDraw tool provided by the Wimley laboratory (Tulane University, New Orleans, LA, USA) (http://www.tulane.edu/~biochem/WW/PepDraw/; accessed on 25 September 2018), and the online I-TASSER server available on the Yang Zhang laboratory website (http://zhanglab.ccmb.med.umich.edu/I-TASSER/; accessed on 25 September 2018) was used to obtain a three-dimensional (3D) image of its secondary structure.

## Figures and Tables

**Table 1:** Antimicrobial activity spectrum of U1-SCRTX-Lg1a.

Microorganism	MIC (μM (μg/mL)) ^1^
Gram-negative bacteria	
*Escherichia coli* SBS363	4.6 (7.6)
*E. coli* D31	4.6 (7.6)
*Pseudomonas aeruginosa* ATCC 27853 *P. aeruginosa* PA14 *Enterobacter cloacae* β-12	1.15 (1.9) 4.6 (7.6) 2.3 (3.8)
Gram-positive bacteria	
*Micrococcus luteus* A270	ND
*Staphylococcus aureus* ATCC 29213 *Bacillus subtilis* ATCC 6633	ND ND
Fungus	
*Aspergillus niger*	ND
Yeasts	
*Candida albicans* MDM8 *Candida krusei* IOC 4559	ND ND

MIC, minimum inhibitory concentration; ND, not detectable (antimicrobial activity not detected in the concentrations assayed). ^1^ The MIC refers to the minimal peptide concentration without visible cell growth in liquid medium.

**Table 2:** Physicochemical parameters of U1-SCRTX-Lg1a.

Net charge	−3
Theoretical isoelectric point (pI)	3.77
Molar extinction coefficient (ε)	51,100 M^−1^ cm^−1^
Aliphatic index	60.62
GRAVY (grand average of hydropathicity)	−0.769 ^1^
Instability index	−11.26 ^2^

Physicochemical parameters were obtained using the ProtParam tool in ExPASy (http://web.expasy.org/protparam/; accessed on 25 September 2018). ^1^ The negative GRAVY value suggests that the peptide is hydrophilic. ^2^ The instability index rates this peptide as stable.
